# Seizure-Control Effect of Levatiracetam on Juvenile Myoclonic Epilepsy and Other Epileptic Syndromes: Literature Review of Recent Studies

**Published:** 2015

**Authors:** Arsalan HASHEMIAGHDAM, Amirsina SHARIFI, Mojtaba MIRI, Abbas TAFAKHORI

**Affiliations:** 1 Iranian center of Neurological research, Imam Khomeini Hospital, Tehran University of Medical Sciences; 2 Neurosurgery department, Imam Khomeini Hospital, Tehran University of Medical Sciences

**Keywords:** Juvenile myoclonic epilepsy, Epileptic syndromes, Levatiracetam, Seizure-control, Side effects

## Introduction

Juvenile myoclonic epilepsy (JME) is the most common idiopathic generalized epileptic syndrome, mostly initiated before adolescence. It is usually preceded by myoclonic jerks (sudden brief involuntary muscle spasm) in the second decade of life. JME may be presented with different types of seizure activity. Simple bilateral myoclonic seizure is the most seen clinical presentation of JME (1). Family history of epilepsy was positive in about 27–44% of patients indicating underlying genetic susceptibility of the disease (2, 3). Typical seizure episodes occur after awakening in the early morning or evening relaxation period (4). Sleep deprivation, fatigue, and alcohol consumption can trigger seizures. Genton et al. proposed that clinicians should consider an early onset epileptic episode as JME in young adults until it is ruled out (4). Myoclonic patterns can be subdivided into essential, physiological, epileptic, and symptomatic. The exact etiology is still unknown. The most probable etiologies are hypoxia, storage disease, toxic-metabolic disorders, drug reactions, and neurodegenerative disorders (5, 6). Recently, five autosomal dominant genetic disorders leading to primary channelopathies have been described. In addition, other susceptible genes for these diseases have been located; however, only 10% of patients showed these changes in their genome (7, 8). Initial screenings that lead to early diagnosis should be used for the management of myoclonus. In complicated cases, further clinical investigations such as enzyme function activity test, histological biopsy, and genetic testing may be needed. Myoclonic activity may originate in different brain areas from sub-cortical areas to brainstem, spinal cord, and the peripheral nervous system with the most common source being the motor cortex. Treatment planning is not achievable unless clinical symptoms of the myoclonus are vividly defined. In the case of failure in for treatment of the underlying etiology, symptom relief should be considered as the treatment goal, though possible side effects and lack of controlled evidence will be challenging issues for physicians (9- 11). Unilateral jerks, generalized tonic-clonic seizures, and focal abnormalities in the EEG can postpone an exact diagnosis that increases misdiagnosis rates (3, 12, 13). However, appropriate disease management would result in preserved life quality in most patients (4, 14, 15). Recently, much research has focused on juvenile myoclonic epilepsy and its phenotypical traits (16). Previous research show juvenile myoclonic seizures include 5–10% of all cases diagnosed with epilepsy. It is estimated that 18% are idiopathic generalized epilepsies. Females are at higher risk of developing this condition (17). 

**Levetiracetam: Pharmacology**

Levetiracetam (LEV) [(S)-α-ethyl-2-oxo-1-pyrrolidine acetamide] is an anti-epileptic drug that has become one of the most prescribed drugs for the management of epileptic syndromes (18-20). Less drug interactions, milder side-effects, and broad-spectrum efficacy make LEV the first choice for many neurologists to control seizures (20). Different mechanisms of actions have made LEV a novel anti-epileptic drug with pharmacologic research not reporting any effect on known neurotransmission mechanisms. Intravenous use of LEV has shown significant success in neonatal and pediatric seizures as well (21-23). LEV has no affinity to GABA (γ-Aminobutyric acid) and glutamatergic receptors without any direct interaction on benzodiazepine binding sites. LEV partially blocks N-type high voltage-activated calcium channels and reduces Ca 2+ release from the neurons. Recently, synaptic vesicle protein 2A (SV2A) was defined as the LEV binding site. The hypothesis of this drug affecting on alpha-amino-3-hydroxy-5-methyl- 4-isoxazole propionic acid (AMPA) receptor channels in mice cortical neurons has been published recently. This recent article revealed that LEV modulates AMPA receptors and changes excitatory post-synaptic function in cortical neurons (11). There are reports that LEV limits epileptogenesis, a process by which an injury into brain stimulates spontaneous seizures (20, 24, 25). 

**Purpose**

We have reviewed some of the most outstanding studies available for analysis in the PubMed and Medline databases about the effect of LEV concerning seizure control. These studies have been dedicated to find the efficacy and tolerability of LEV in different therapeutic regimens, different epileptic syndromes, as well as in different age groups. The side effects of LEV are a great matter of concern and they were investigated in some studies are indicated. 

**Levetiracetam in different epileptic syndromes:**

Tonekaboni SH et al. administered LEV as an add-on therapy for 45 children aged 0.6–15 years (mean age 5.9 years). These children were known to have JME that is not responding to most conventional drugs. The starting dose was 20mg/kg/day and was increased at one-week intervals by 10mg/kg/day to a maximum dose of 60mg/ kg/day, if it was necessary. In the 12 week period of follow up, four children (8.7%) became seizure free, four more children (8.7%) indicated an increase in seizure frequency, and in 8 (17.4%) and 13 (28.3%) patients, seizure frequency decreased by 75–99% and 50–74%, respectively. They concluded that LEV was effective in 54.3% of patients and decreased seizure frequency to at least 50% of the baseline seizure frequency. They also found that sex, age, duration of disease, type, and cause of seizure, EEG, and imaging data, and type of epileptic syndrome had no significant correlations with improvement in the epileptic course. Finally, they claimed that LEV is an effective add-on therapy in patients with refractory epilepsy (26). Magaudda et al. investigated the effects of LEV in 13 patients (2 women and 11 men, aged 14–52 years; mean 36.5 years) with Unverricht-Lundborg Disease (ULD). This condition is inherited by autosomal recessive mutation in the Cystatin B gene and progressive myoclonus epilepsy is a major symptom. LEV was prescribed in addition to previous medication in dosages of 2,000–4,000 mg/d for 0.5–26 months (mean 13.8 months), while the patients were seen by the physician at regular 3–6 month intervals. They examined seizure frequency by using simplified myoclonus rating score, EEG tracings, treatment regimens, and clinical examination performed at the time of the follow-ups. They have also screened unexpected complications, social status, and general well-being. Among this group, only one patient quit the trial because of side effects like drowsiness, restlessness, and lack of symptom relief. Others showed no side effects. All patients reported a decrease in myoclonus but 8 patients had improvements on a rating scale that was from 3.1–2.4 (p = 0.01). There was a correlation between the duration of the disease and the effect of LEV. LEV was more efficient in patients with lower duration of the disease. For example, 5 patients who did not show any progress in rating score had a mean duration of disease of 30 years versus 19.3 years in 8 patients who showed minor improvements. Interestingly, in patients with a previous intake of high-dose Piracetam (PIR), there was a worsening of symptoms. This evidence signaled patients to continue PIR at a lower or at the same dose. Patients who have never obtained PIR showed a vivid improvement with LEV. Although LEV was given as an additional medication, the writers of this manuscript believe that it might be more effective if administered earlier. They reported a combination of low dose PIR and LEV as a practical solution and considered LEV as a major treatment option in early stages of ULD (27, 28).

**Table 1:** Effect of LEV on various epileptic syndromes

	**Starting Dose**	**Type of Epileptic Syndrome **	**Duration of Treatment**	**Rate Of Response**	**Rate Of Seizure Free Patients**	**Age**	**Complications**
**Tonekaboni et al**	20 mg/kg/day	JME	3 Months	54.30%	8.70%	Mean: 5.9 years	none
**Magaudda et al**	2,000–4,000 mg/d	progressive Myoclonus Epilepsy	13.8 Months	92.30%	61%	Mean: 36.5 years	drowsiness, restlessness, and lack of symptom relief
**Maw J et al**	10–15 mg/kg/day	various epileptic syndromes	1.5 Months	69%	Not Reported.	Mean: 7 years	behavioral disturbances, excessive sleepiness, and worsening of symptoms
**Verotti et al**	250 mg	JME	6 Months	90.60%	46.80%	Mean: 13.1 years	none
**Verotti et al**	250 mg	JME	12 Months	90.60%	90.60%	Mean: 13.1 years	none
**Lagae et al**	10 mg/kg/day	various epileptic syndromes	3 Months	50% (mono-therapy ) 81% (add-on therapy)	none among JME patients	0.5–16years	tiredness (dose dependent), anorexia , positive behavior changes, and alertness

**Efficacy and possible side effects of LEV in pediatrics**

In childhood epilepsy, the negative effects of antiepileptic drugs on cognitive functions during

development of the brain are as important as seizure control. Lagae et al. indicated that 77 children (.5–16 years) with different etiologies of childhood epilepsy with both partial and generalized seizure attacks were divided into a trial of “mono therapy” or “add-on therapy” with LEV, as if they met the inclusion criteria for each group. The effect of LEV on behavior and alertness was one of the primary goals of this study. In each group, LEV was started at 10mg/kg/day in two equal dosages and was increased by 10mg/kg weekly to a maximum of 60 mg/kg/day according to efficacy and tolerability. Previous anti-epileptic medications were administered simultaneously with the first 12 weeks of medications. Crucial data were collected using patients diaries and history to calculate the frequency of seizures.

Overall quality of life representing both efficacy and tolerability was scored by parents on a scale of 0–10, with 10 being the best overall quality of life. There was a median reduction of 50% and 81% in the add- on group and the mono-therapy group, respectively. In both groups, there were patients who became seizure free. 

LEV has not proved to be a superior or an inferior choice of anti-epileptic drug in generalized or partial seizures in different epilepsy syndromes or in combination therapy with other anti-epileptic drugs. The main adverse effect of LEV was tiredness and it was seen only in the add-on group. This adverse effect was dose-dependent and 4/6 of patients with tiredness had taken >45mg/kg/ day. In the mono-therapy group, anorexia was seen in only one child. In about 1/4 of the patients, there was a positive effect on alertness and behavior. This means that children were more capable of communicating with caregivers and were better handled and structured. This effect was not because of seizure reduction as there was 

a reduction of less than 50% in 7/18 and 7/16 of patients who showed increased alertness and better behavior, respectively. Quality of life was reported to be better in the mono-therapy group as parents rated median score of 8 versus 6 in the add-on group. They believed that LEV could be beneficial for both partial and generalized seizures (29-33).

Maw J investigated the efficacy and tolerability of LEV in children aged 10 years and younger as an add-on therapy. They gathered initial data from 26 patients who suffered from different epilepsy syndromes

(west syndrome: 1; Lennox-Gastaut Syndrome: 4; Symptomatic generalized epilepsy: 10; Cryptogenic partial epilepsy: 2; Symptomatic partial epilepsy: 9), with a median age of 7 years (ranged from 14 months to 10 years). All patients previously received antiepileptics with a median number of 4 (range, 1–9). LEV was started at once daily dosage of 10–15mg/kg/day and was increased every two weeks if tolerated (early reported side effects were labeled as tolerable). The mean maintenance daily dose was 36.9 mg/kg/day (range, 13.5–68.5). The most common adjunctive anti-epileptics were sodium valproate, lamotrigine, carbamazepine, and one of the benzodiazepines. Best response to LEV was seen in patients with partial-onset seizures and the worst was seen in myoclonic seizures patients. Two children with PEHO (Progressive encephalopathy with Edema, Hypsarrhythmia (and Optic atrophy syndrome) and

Alpers’ disease showed no response to LEV, and it was suspended after 6 and 9 weeks, respectively. In three patients, pre-existing myoclonic seizures worsened after administration of LEV. Two of these three other seizure types, nevertheless showed minor improvements. One patient claimed adverse effects of excessive sleepiness as the reason for the suspension of the trial. According to these findings, investigators view LEV to be a well-tolerated anti-epileptic with a negligible amount of adverse effects. However, there is evidence reporting a higher rate of behavioral disturbances in children with

neurological disorders after initiation of LEV in the treatment (34). It might be useful as an adjunct therapy of partial seizures in young children with refractory epilepsy. It is stated that LEV has little effect on myoclonic seizures (35, 36).

Primary generalized epilepsy (PGE) is routinely treated with valproic acid. More recently, lamotrigine, topiramate, gabapentin, carbamazepine, phenytoin, tiagabine, and vigabatrin are no longer prescribed as they may aggravate absence seizures or myoclonic jerks (37). Jeffrey Cohen et al used LEV in three patients with PGE, refractory to other anti-epileptic drugs (AED). Three similar patients almost had the same medical history for convulsions and their physical and neurological examinations were normal as were brain CT and MRI but EEG patterns matched the typical pattern of PGE. Clinical diagnosis of PGE could be challenging due to incomplete history of myoclonic jerks and absence attacks. In this regard, EEGs could be helpful for accurate classification of seizures. These patients became seizure-free in effective dosages ranged from 1,250–3,000 mg/day, while other AEDs were tapered down. In comparison to other AEDs, LEV binds to serum protein less and does not interfere with other AEDs. In addition, LEV has no effect on hepatic enzyme levels. One of the practical advantages of LEV is that it could be started at a therapeutic dose of 1,000 mg/day and increased to 3,000mg/day within four weeks Unlike LEV, lamotrigine requires slow titration that may require weeks to achieve the preferred dosage. 

Although this study had a small group of patients to investigate, because of precise, complete documented

clinical, and paraclinical information; it can be relied on and is considered a reliable source. This study suggested that LEV can be safe and effective choice for patients with PGE and especially for those who are refractory to other AEDs (38).

As we have previously discussed, the main side effect of LEV is somnolence but Eric H. Kossoff indicated that four patients had extreme behavioral changes and psychosis were reported. The first case was a five-year-old girl with a history of multifocal epilepsy and with mild mental retardation. She had a ketoge nic diet as her only treatment before starting LEV. She was given 250 mg of LEV twice a day. She began having visual hallucination of spiders two weeks after starting LEV and was seizure free over the prior two-week period. After abrupt discontinuation of LEV, her symptoms were resolved and did not recur. 

The second case was a 13-year-old boy with history of refractory complex partial seizures and left lobe sharp waves on his EEG. He also had a learning disability and oppositional behavior toward authority figures. About 3 months after beginning LEV mono-therapy at 500 mg twice a day, he had an acute onset of auditory and visual hallucinations of a female person in his room. His psychosis was resolved as LEV was dismissed.

The third case involved a 16-year-old girl with a right temporoparietal lobe dysembryoplastic neuroepithelial tumor. She was on carbamazepine mono-therapy for further evaluation of surgical treatment. She had history of complex partial epilepsy and Weschler full-scale IQ of 76 (borderline intelligence). She was given 500 mg twice a day as she was preparing for surgery. On day 2 of LEV administration, she became acutely agitated and attempted to run away from home. An EEG showed the right temporal lobe slowing but no epileptiform discharges. Psychiatry approved drug-induced psychosis

and 3 days later, she was almost back to her status. The fourth case 4 was a 17-year-old girl with a history of mild cognitive impairment, depression, and left temporal lobe epilepsy related to head trauma from 10 years ago. She was on carbamazepine at a dose of 800 mg three times a day and LEV was added at 1,000mg twice a day in two week period titration. After 30 days of administration, she started to have headaches, lethargy, and auditory hallucination of a sound outside her bedroom telling her to sing and dance. LEV was discontinued and the sound was gone. One-month later medication started at a lower 

dosage of 500 mg twice a day without any psychotic symptoms recurrence. Possible causes for these adverse effects may be a rapid initiation of the dose, high total dose, young age, and underlying cognitive or behavioral abnormalities. Finally, the writers suggested slow initiation of 10mg/kg/day and increased to 20mg/kg/day over a four week-period, especially in patients with comorbid neurobehavioral abnormalities (39).

Albert Verrotti et al. conducted research on the efficacy and tolerability of LEV as mono-therapy in patients with JME. In this multicenter, prospective, long-term, and open label treatment, 32 patients (20 females and 12 males) with JME were included and median age was 13 years and three months (SD, 7 years and 11 months). Newly diagnosed patients and EEG-specificw abnormalities (3–6 spike/poly-spike slow wave discharge) were enrolled in the study. LEV was started at 250 mg once each evening and escalated gradually to a maximum dose that ranged from 1,000–2,500 mg/ daily. All patients were followed up at 6 and 12 months after and patient diaries and history were taken for the number and type of seizures, adverse effect, response to LEV treatment, as well as its outcome. Physical and neurological examinations, laboratory assessment of CBC, UA, blood Cr and BUN, amylase, AST and ALT and electrocardiography was performed. At the 6-month follow up, 15 patients were seizure free (100% seizure control) and 14 were responders (>50% reduction of seizures) and 3 were in marginal effects group (<50% reduction of seizures). At the 12 month follow up, all patients were seizure free except those in the marginal group at the 6 month follow up. There were no abnormal findings in follow up examination tests and there were no side effects reported. This study revealed that LEV could be effective in JME as a first line mono-therapy (11, 40).

**In conclusion, **LEV is a novel anti-epileptic drug that is chemically related to a previous known drug,

piracetam, but with different mechanism of action. It appeared to be effective in decreasing myoclonic

seizures with different etiologies, which varied from epileptiform EEG abnormalities to refractory generalized epilepsy in children or adults and idiopathic generalized epilepsy, juvenile myoclonic seizures, and even epileptic syndromes like Unverricht-Lundborg Disease. This new medication can be used even as mono- or add-on therapy to previous anti-epileptic drugs but greater positive effects were observed in patients who took LEV as mono-therapy. One of the amazing advantages of LEV is that it can be started at a high therapeutic dosage and is well tolerated. The median starting dosage varied according to patients underlying disease, age, and disease severity but it can range from 250 mg–1,000 mg daily in a single or a double dose. Median maintenance dosage can vary from 2,000–3,000 mg daily. Myoclonic seizure responds dramatically and different studies have reported a seizure absence of > 50% in more than 90% of patients in both mono- or add-on therapy, but a greater number are seizure free in the mono-therapy group and JME. However, physicians cannot ignore the evidence of behavioral side effects in pediatric patients. Most patients who showed worsening of the condition or had unchanged clinical course also had refractory generalized epilepsy. It is well-known in the literature that sodium valproic acid is the drug of choice for newly diagnosed myoclonic seizures but according to previous studies it was demonstrated that the side effects of LEV, mainly tiredness and somnolence, are by far less and much safer compared to valproic acid. This is the case especially in female patients mainly because of the teratogenicity effects of valproic acid in pregnancy and because of polycystic ovarian syndrome in females of reproductive age. 

Further investigations should be conducted on large sample groups to evaluate its efficacy and tolerability

in specific age groups and clinical conditions such as pregnancy.

## Author Contribution

Hashemiaghdam: Main Author, Contribution to Conception and design, Analysis and interpretation, Data collection, Writing the article,Critical revision of the article, Final approval of the article ,Statistical analysis, Overall responsibility Sharifi: Main Author, Contribution to Conception and design, Analysis and interpretation, Data collection, writing the article, Critical revision of the article Dr. Miri: Co-Author contribution to Conception and design, Critical revision of the article, Final approval of the article Dr. Tafakhori: Corresponding author, contribution to Conception and design, Analysis and interpretation, Critical revision of the article, Final approval of the article, Statistical analysis, Overall responsibility.
